# Serum concentrations of kynurenines in adult patients with attention-deficit hyperactivity disorder (ADHD): a case–control study

**DOI:** 10.1186/s12993-015-0080-x

**Published:** 2015-11-05

**Authors:** Tore Ivar Malmei Aarsland, Elisabeth Toverud Landaas, Tor-Arne Hegvik, Arve Ulvik, Anne Halmøy, Per Magne Ueland, Jan Haavik

**Affiliations:** Department of Biomedicine, University of Bergen, Jonas Lies vei 91, 5009 Bergen, Norway; K.G. Jebsen Centre for Research on Neuropsychiatric Disorders, University of Bergen, Bergen, Norway; Bevital A/S, Bergen, Norway; Division of Psychiatry, Haukeland University Hospital, Bergen, Norway; Section for Pharmacology, Department of Clinical Science, University of Bergen, Bergen, Norway

**Keywords:** Attention-deficit hyperactivity disorder, Kynurenine, Tryptophan, Vitamin B, Cotinine, Inflammation, Biomarker

## Abstract

**Background:**

The essential amino acid tryptophan is catabolised mainly through the kynurenine pathway. Altered circulating levels of kynurenines have been reported in chronic inflammatory conditions and in several neuropsychiatric disorders, including depression and schizophrenia. Candidate gene studies suggest that genes related to the kynurenine catabolism may be associated with attention-deficit hyperactivity disorder (ADHD). Additionally, ADHD patients often report comorbid depression or anxiety. In this study we investigated serum levels of kynurenines in Norwegian adult ADHD patients and adult controls.

**Methods:**

We compared serum levels of tryptophan and the seven tryptophan metabolites kynurenine, kynurenic acid, anthranilic acid, 3-hydroxykynurenine, xanthurenic acid, 3-hydroxyanthranilic acid and quinolinic acid in 133 adult patients with ADHD and 131 adult controls (18–40 years). Riboflavin (vitamin B2), total vitamin B6 and the nicotine metabolite cotinine were also measured. Serum samples were analysed using mass spectrometry. Patients and controls reported comorbid disorders and past (childhood) and current ADHD symptoms using the Wender Utah Rating Scale (WURS) and the Adult ADHD Self-report Scale (ASRS). Logistic regression was used to calculate odds ratios for having an ADHD diagnosis for different serum levels of each metabolite. In addition, we used Spearman’s correlation analysis to investigate the correlation between serum levels of tryptophan and kynurenines and ADHD symptom scores.

**Results:**

Lower serum concentrations of tryptophan [odds ratio 0.61 (95 % confidence interval 0.45–0.83)], kynurenic acid [0.73 (0.53–0.99)], xanthurenic acid [0.65 (0.48–0.89)] and 3-hydroxyanthranilic acid [0.63 (0.46–0.85)], and higher levels of cotinine [7.17 (4.37–12.58)], were significantly associated with ADHD. After adjusting for tryptophan levels, only 3-hydroxyanthranilic acid and cotinine remained significant. Lower levels of tryptophan and kynurenine were also found to be correlated with higher total ASRS score and higher total WURS score, when adjusting for smoking and age.

**Conclusions:**

Our results suggest that there may be differences in serum levels of tryptophan and kynurenines between adult ADHD patients and adult controls. Although our findings do not suggest a chronic immune activation in ADHD, the underlying mechanisms and possible clinical implications of the differences should be further explored.

## Background

Attention-deficit hyperactivity disorder (ADHD) is a neurodevelopmental disorder characterised by inattention, hyperactivity and impulsivity and has a pooled prevalence rate of about 2.5 % in the adult population [1]. The symptoms are often severe and may cause serious difficulties in the daily life of affected individuals [2]. The disorder often coexists with other neuropsychiatric disorders like depression and bipolar disorder, with which it also shares symptoms [3, 4]. The aetiology of ADHD is complex and is most likely explained by the combined impact of many environmental and genetic factors [5, 6]. In a recent study on ADHD and its relation to low birth weight, it was suggested that genetic variants in the kynurenine pathway might contribute to ADHD symptom severity [7].

**Fig. 1:**
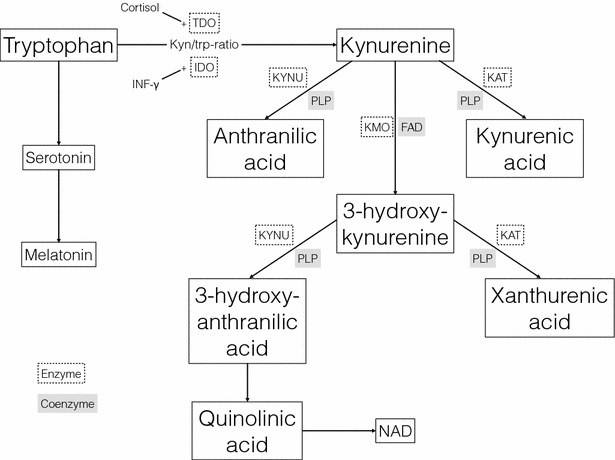
The kynurenine pathway of tryptophan catabolism. *INF-γ* interferon gamma, *TDO* tryptophan 2,3-dioxygenase, *IDO* indole 2,3-dioxygenase, *KYNU* kynureninase, *KAT* kynurenine aminotransferase, *KMO* Kynurenine mono-oxygenase, *PLP* pyridoxal 5′-phosphate (vitamin B6), *FAD* flavin adenine dinucleotide (vitamin B2), *NAD* nicotinamide adenine dinucleotide

The kynurenine pathway (Fig. 1) constitutes the major route for catabolism of the essential amino acid tryptophan [8]. More than 90 % of tryptophan is catabolised to kynurenine, mainly in the liver by the tryptophan specific enzyme tryptophan 2,3-dioxygenase (TDO), but also in lungs, kidneys, spleen, placenta and blood by the enzyme indole 2,3-dioxygenase (IDO) [9]. In the brain, the catabolism of tryptophan to kynurenine is driven by TDO and IDO located in astrocytes and microglia [9]. The activity of TDO depends on the concentration of tryptophan and is stimulated by high levels of cortisol, while IDO activity is enhanced mainly by pro-inflammatory cytokines, such as interferon-γ, and reduced by the anti-inflammatory cytokine interleukin 4 [9]. Kynurenine is further converted into kynurenic acid (KA), by kynurenine aminotransferase (KAT I, II, III), or by kynurenine-3-monooxygenase (KMO) into 3-hydroxykynurenine (HK), which is metabolised to 3-hydroxyantranilic acid (HAA) and eventually quinolinic acid (QA) [9]. These steps through the kynurenine pathway are dependent on the coenzymes pyridoxal 5′-phophat (PLP), the active form of vitamin B6 [8, 10], and flavin adenine dinucleotide (FAD), the active form of riboflavin (vitamin B2) [10]. Thus, diet may influence tryptophan metabolism either directly or via vitamin levels. Moreover, serum levels of tryptophan and vitamin B2 and B6 may be affected by smoking [11, 12].

KA and QA have neuroactive properties and exert multiple effects in the central nervous system. KA is an antagonist of the glutamatergic NMDA receptor and the cholinergic nicotinic α7-receptor and as such an endogenous protector against excitotoxicity [9]. High cerebrospinal fluid (CSF) levels of KA have been associated with cognitive dysfunction both in animals [13] and in humans, including patients with schizophrenia [14, 15]. Reduced plasma levels of KA have been reported in depression [16] and in patients with bipolar disorder [17]. QA is an agonist of the NMDA receptor and may cause excitotoxicity [18]. High levels of QA have been found in several neurodegenerative diseases such as Huntington’s disease and Alzheimer’s disease [18].

ADHD often coexists with other neuropsychiatric disorders that have been associated with altered levels of metabolites of the kynurenine pathway, such as major depression and bipolar disorder [3, 4]. Furthermore, known environmental risk factors for ADHD, such as preeclampsia, postnatal infection and malnutrition, may involve abnormal tryptophan catabolism [19, 20]. Neuropsychological deficits, for example in executive functioning, are often found in ADHD [21], and are thought to be related to a hypofunctional dopamine system [22]. Tryptophan metabolites can modulate several neurotransmitter systems, including dopaminergic transmission [9]. Moreover, in addition to kynurenine, tryptophan is also the precursor of serotonin (5-hydroxy-tryptamine, 5-HT), a neurotransmitter that has been suggested as an important agent in several neuropsychiatric disorders including ADHD [23].

In this study we sought to compare serum levels of kynurenines between adult ADHD patients (N = 133) and adult controls (N = 131) and also across continuous scales of self-reported past and present ADHD symptom scores (N = 264).

## Methods

### Participants

ADHD patients and controls were recruited as part of the Norwegian study “ADHD in adults in Norway: from clinical characterization to molecular mechanisms”, initiated at the University of Bergen in 2004. Most patients were recruited by responding to invitation letters sent to people listed in a Norwegian national registry of adult ADHD patients. Some patients were recruited directly from psychiatrists or outpatient clinics, as described earlier [24]. All patients had been previously diagnosed with ADHD using either DSM-IV [25] or ICD-10 [26]. The ICD-10 criteria were adapted to the DSM-IV criteria by allowing the inattentive subtype as sufficient for the ADHD diagnosis, and by accepting the coexistence of other neuropsychiatric disorders as long as they appeared after the criteria of ADHD were fulfilled. For the present study, only participants from whom we had access to blood samples, who were between 18 and 40 years, were of Norwegian ancestry, and who volunteered to participate after receiving oral and written information about the project were included (N = 264). The patient group consisted of 133 adults (71 females, 62 males) and the control group consisted of 131 students (75 females, 56 males) recruited locally at the University of Bergen.

### Measures

The majority of blood samples were collected locally at the Haukeland University Hospital campus in Bergen. Some samples were also collected by primary care physicians in Bergen and at other locations in Norway. Fasting was not required before blood sampling. Blood samples were collected in serum tubes with gel separator. Samples collected outside the hospital were transported by mail to the laboratory. During transport, samples were kept at ambient temperature. Immediately upon arrival at the laboratory serum was separated and stored at −80 °C. All samples were visually inspected and samples with signs of haemolysis or degradation were excluded (N = 2). To avoid batch effects, control and patient samples were analysed together. Every second sample was from either controls or patients. Tryptophan and the seven kynurenines, kynurenine, kynurenic acid (KA), anthranilic acid (AA), xanthurenic acid (XA), 3-hydroxykynurenine (HK), 3-hydroxyanthranilic acid (HAA) and quinolinic acid (QA), as well as riboflavin (vitamin B2), B6 vitamers pyridoxal (PL) and pyridoxal-5′-phosphate (PLP), and the nicotine metabolite cotinine [27], were measured using stable isotope dilution liquid chromatography-tandem mass spectrometry, as described [28]. All biochemical analyses were performed by Bevital AS (http://www.bevital.no), Bergen.

All participants completed questionnaires with information on mental health, education, occupational status, and lifetime psychiatric co-morbidity. The latter was assessed by questions like “Have you ever experienced significant anxiety and/or depression?” and “Have you ever had problems with alcohol?” [3]. For assessment of current ADHD symptoms, all participants filled in the 18-item Adult ADHD Self-report Scale (ASRS) consisting of 9 questions specific to symptoms of inattentiveness and 9 questions on symptoms of hyperactivity and impulsivity, all with a 0–4 point Likert scale of symptom severity (0 = never/seldom, 4 = very often) [29]. In addition the 25-item version of the Wender Utah Rating Scale (WURS) was used for retrospective assessment of ADHD symptoms in childhood, each question with a Likert scale of 0–4 indicating the severity of the symptom (0 = not at all/very slightly, 4 = very much) [30].

### Statistical analyses

To explore the material, age and ADHD symptom scores in the patient and control group were compared using Student’s *t* test, while χ^2^ test were used for comparing the categorical variables sex, alcohol and drug abuse, comorbid disorders and subgroups of ADHD. Total vitamin B6 level was calculated as the sum of PL and PLP [31]. Kynurenine/tryptophan ratio (KTR), a marker of immune activation, was calculated as the serum concentration of kynurenine divided by the concentration of tryptophan, multiplied with 1000. About 80 % of the biochemical variables were non-normally distributed, and levels in patients and controls were therefore investigated using Mann–Whitney U tests. Spearman’s correlations for biochemical variables, age and sex were used to further explore the data.

For use in logistic regression all biochemical variables were split in tertiles, except for cotinine which was divided in three categories corresponding to the serum level of cotinine in non-smokers (<80 nmol/l), moderate smokers (80–1000 nmol/l) and heavy smokers (>1000 nmol/l) [32]. These three-way categorisations were chosen to assess possible effects of low, medium and high levels of the biochemical variables/smoking. Blood cotinine can also be derived from nicotine in smokeless tobacco/snuff tobacco. Although we could not discriminate between different sources of nicotine, we considered the contribution from smokeless tobacco to be small in our study. At the time of sample collection, regular smoking was much more common than smokeless tobacco consumption. Some users also combine several sources of tobacco/nicotine (https://helsedirektoratet.no/english/tobacco-control). Logistic regression with patient status (ADHD yes/no) as outcome was performed for each biochemical variable in two models, one with sex as covariate and another adjusting for both sex and tryptophan. Odds ratio (OR) with 95 % confidence interval (CI) for ADHD was calculated per tertile of each metabolite (per category in the case of cotinine).

There was a greater age span in the ADHD group than in the control group, and it is known that tryptophan levels may decrease with age. Logistic regressions were therefore performed with two age subgroups of ADHD patients as outcome for each biochemical variable with adjustment for sex. The first age group, 19–33 years (N = 97), corresponded to the age span of the control group, while the second group contained the patients with no age-matched controls (34–40 years, N = 36). Associations between biochemical variables and patients’ comorbid disorders, i.e. bipolar disorder and anxiety/depression, were also investigated using logistic regressions adjusted for sex. A last logistic regression analysis was performed to explore the effect of current treatment with central stimulants.

Partial correlation analyses (Spearman’s) were used to explore associations between ADHD symptom severity and levels of biochemical variables, while adjusting for smoking (cotinine level) and age. The analyses were performed in all participants and for patients and controls separately.

Student’s t test, χ^2^ test, Mann–Whitney U test and regular Spearman’s correlation analysis were performed in Statistical Package for Social Sciences version 22 (IBM Corp., Armonk, New York) for Apple OSX. All regression models as well as partial Spearman’s correlation were performed using R (The R Project for Statistical Computing) for Apple OSX. All p values were two-sided, and statistical significance was defined at the 5 % level.

### Ethics

All patients gave written informed consent and the protocol was approved by the Regional Committee for Medical Research Ethics, REK Vest (IRB #3 (FWA00009490, IRB00001872)).

## Results

### Clinical data

There were 264 participants included in this study, 133 adult ADHD patients and 131 adult controls. A comparison of clinical data is shown in Table 1. Women were slightly overrepresented in both groups (53 and 57 %). The median age of the patient group was significantly higher than that of the control group (28.0 versus 22.5 years). In the patient group, 65.1 % reported a lifetime history of significant anxiety or depression, while this was reported by only 3.8 % of the controls. Among the ADHD patients, 9.9 % also reported having a comorbid bipolar disorder, compared to none in the control group. There was a significant difference in the number of smokers, with 66.2 % of the patients having a serum cotinine level of >80 nmol/l, compared to only 12.2 % of the controls. As expected, median childhood ADHD symptom scores (WURS) and median present ADHD symptom scores (ASRS) were much higher in the patient group (60 and 47 versus 12 and 22 in the control group). According to standard cut-offs for ASRS, 6.2 % of the controls screened positive for ADHD.

**Table 1:** Clinical and biochemical data

	ADHD	N	Control	N	p value
		133		131	
Female, N (%)	71 (53.4)	133	75 (57.3)	131	0.52^a^
Age (range 18–40), median (SD)	28.0 (6.5)	133	22.5 (2.8)	131	<0.001***^b^
Alcohol problems, N (%)	24 (19.2)	125	1 (0.8)	131	<0.001***^a^
Problems with illicit drugs, N (%)	34 (27.0)	126	0 (0.0)	131	<0.001***^a^
Serious anxiety and/or depression, N (%)	82 (65.1)	126	5 (3.8)	126	<0.001***^a^
Bipolar disorder, N (%)	12 (9.9)	121	0 (0.0)	131	<0.001***^a^
Moderate smokers^d^, N (%)	51 (38.3)	133	12 (9.2)	131	<0.001***^a^
Heavy smokers^d^, N (%)	27 (27.8)	133	4 (3.1)	131	<0.001***^a^
Total WURS (range 0–100), median (SD)	60.0 (16.9)	118	12.0 (10.4)	129	<0.001***^b^
Total ASRS (range 0–72), median (SD)	47.0 (11.6)	123	22.0 (7.7)	128	<0.001***^b^
Combined type^e^, N (%)	65 (52.8)	123	3 (2.3)	128	<0.001***^a^
Hyperactive type^e^, N (%)	3 (2.4)	123	1 (0.8)	128	0.32^a^
Inattentive type^e^, N (%)	31 (25.2)	123	4 (3.1)	128	<0.001***^a^
Tryptophan^f, i^	77.3 (68.1–92.3)	133	83.6 (76.6–94.0)	130	0.004**^c^
Kynurenine^f, i^	1.51 (1.29–1.78)	133	1.57 (1.40–1.76)	130	0.15^c^
Kynurenine/tryptophan ratio (KTR)^g, i^	19.0 (16.5–21.6)	133	18.4 (16.7–20.8)	130	0.46^c^
3-hydroxykynurenine (HK)^h, i^	31.8 (22.2–39.4)	131	30.6 (26.1–28.1)	130	0.43^c^
Kynurenic acid (KA)^h, i^	38.9 (30.9–52.2)	131	46.7 (34.0–57.5)	130	0.03*^c^
Xanthurenic acid (XA)^h, i^	14.2 (11.0–22.4)	131	18.9 (13.7–24.7)	130	0.004**^c^
Anthranilic acid (AA)^h, i^	19.4 (13.2–24.3)	131	17.6 (14.9–22.8)	130	0.52^c^
3-hydroxyanthranilic acid (HAA)^h, i^	29.8 (20.0–40.1)	131	35.4 (29.2–46.5)	130	<0.001***^c^
Quinolinic acid (QA)^h, i^	303 (258–387)	131	306 (274–364)	130	0.76^c^
Riboflavin (Vit. B2)^h, i^	18.1 (12.7–25.9)	131	19.9 (16.1–28.3)	130	0.02*^c^
Total vitamin B6^h, i^	58.5 (41.8–83.9)	131	67.1 (49.0–88.4)	130	0.05*^c^
Cotinine^h, i^	678 (2.48–1088)	131	1.2 (0.55–6.06)	130	<0.001***^c^

Significance <0.001: ‘***’, <0.01: ‘**’, <0.05: ‘*’

^a^χ^2^ test

^b^Student’s T test

^c^Mann–Whitney U test

^d^Calculated based on serum cotinine levels: 80–1000 nmol/l: moderate smoker, >1000 nmol/l: heavy smoker

^e^Calculated based on ASRS scores: A score of 21 or higher on the nine questions on inattentiveness is indicative of inattentive type, while a score of 21 or higher on the hyperactivity/impulsive questions is indicative for hyperactive/impulsive type. Combined type requires a score of 21 or higher on both subscales

^f^μmol/L

^g^μmol/μmol

^h^ nmol/L

^i^Median (25–75 percentile)

Among patients for whom we had information on medication status, 113 patients (out of 123, 92 %) had a history of central stimulant treatment. When the blood samples were collected, 91 patients (out of 114, 80 %) still received such treatment. Twenty-five patients were currently using anti-depressants.

### Biochemical data

#### Correlations

All kynurenine metabolites were positively correlated to kynurenine levels [Spearman’s rho (r): 0.24–0.58] (Table 2). Tryptophan levels showed a moderate correlation with KA, XA and HAA, (r: 0.34–0.41), and a weak correlation with HK and QA (r: 0.14, 0.18). KA, XA, HAA and QA showed positive correlation with both vitamin B2 (r: 0.15–0.27) and total vitamin B6 (r: 0.18–0.24). All correlations were statistically significant (p < 0.05).

**Table 2:** Unadjusted Spearman’s correlations

	Trp	Kyn	KTR	HK	KA	XA	AA	HAA	QA	Vit. B2	Vit. B6	Cot	ADHD status
All (N = 264)													
Age	−0.24*	−0.05	0.13*	−0.13*	−0.13*	−0.26*	0.09	−0.27*	−0.10	−0.04	−0.23*	0.26*	0.47*
Sex	0.19*	0.18*	0.01	−0.11	0.21*	0.01	0.08	−0.04	−0.03	−0.14*	0.11	0.15*	0.04
Trp		0.49*	−0.46*	0.14*	0.34*	0.41*	0.10	0.34*	0.18*	0.17*	0.32*	−0.13*	−0.18*
Kyn			0.48*	0.39*	0.48*	0.24*	0.36*	0.32*	0.58*	0.16*	0.19*	−0.04	−0.09
Vit. B2			−0.06	0.02	0.15*	0.23*	0.11	0.15*	0.27*		0.32*	−0.13*	−0.14*
Vit. B6			−0.12	−0.12	0.22*	0.20*	0.03	0.24*	0.19*			−0.16*	−0.12*
Cotinine			0.05	−0.06	−0.08	−0.16*	0.02	−0.20*	−0.03				0.52*
ADHD (N = 133)													
Age	−0.30*	−0.08	0.22*	−0.26*	−0.11	−0.31*	0.05	−0.27*	−0.08	0.22*	−0.16	−0.04	
Sex	0.15	0.14	0.03	−0.04	0.08	0.03	−0.02	0.09	−0.00	−0.18*	0.09	0.13	
Trp		0.55*	−0.41*	0.21*	0.34*	0.48*	0.01	0.35*	0.32*	0.18*	0.40*	−0.07	
Kyn			0.46*	0.45*	0.50*	0.29*	0.32*	0.39*	0.64*	0.17	0.21*	0.07	
Vit. B2			−0.03	−0.01	0.15	0.29*	0.10	0.15	0.30*		0.32*	−0.13	
Vit. B6			−0.17	−0.01	0.28*	0.26*	−0.03	0.28*	0.24*			−0.27*	
Cotinine			0.08	0.03	−0.11	−0.19*	−0.03	−0.08	−0.01				
Control (N = 131)													
Age	0.10	0.10	−0.01	0.05	0.01	−0.03	0.10	−0.06	−0.10	−0.19*	−0.24*	0.14	
Sex	0.28*	0.25*	−0.03	−0.19*	0.36*	−0.01	0.20*	−0.11	−0.08	−0.06	0.16	0.20*	
Trp		0.41*	−0.48*	0.04	0.31*	0.27*	0.24*	0.19*	−0.02	0.13	0.21*	0.06	
Kyn			0.55*	0.31*	0.46*	0.17	0.42*	0.16	0.50*	0.10	0.13	0.05	
Vit. B2			−0.10	0.03	0.09	0.12	0.13	0.03	0.21*		0.33*	0.06	
Vit. B6			−0.06	−0.26*	0.14	0.11	0.12	0.14	0.11			0.14	
Cotinine			−0.02	−0.11	0.17	0.03	0.09	−0.10	−0.03				

Significance: <0.05 ‘*’

*Trp* tryptophan, *Kyn* kynurenine, *KTR* kynurenine/tryptophan ratio, *HK* 3-hydroxykynurenine, *KA* kynurenic acid, *XA* xanthurenic acid, *AA* anthranilic acid, *HAA* 3-hydroxyanthranilic acid, *QA* quinolinic acid, *Cot* cotinine

There was a significant inverse correlation between age and tryptophan in this material as a whole (r: −0.24) and in the patient group (r: −0.30) in particular. A positive, although non-significant, correlation between age and tryptophan was also found in the control group (r: 0.10). There was a significant but weak inverse correlation between cotinine and tryptophan (r: −0.13), XA (r: −0.16), HAA (r: −0.20), riboflavin (r: −0.13) and total vitamin B6 levels (r: −0.16) when including all participants.

#### Metabolite levels according to ADHD status

**Fig. 2:**
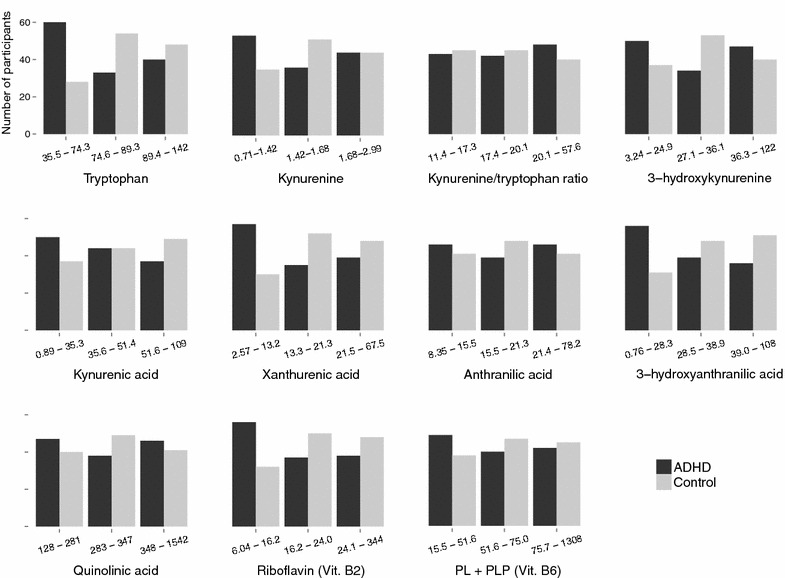
Distribution in tertiles of biochemical variables. Each pair of columns contains a third of the participants. All participants are included (N = 264). ADHD patients are strongly represented in the lower concentrations of several metabolites, including tryptophan, XA, HAA and riboflavin. Controls are in majority in the middle concentrations of all metabolites except for KA

**Fig. 3:**
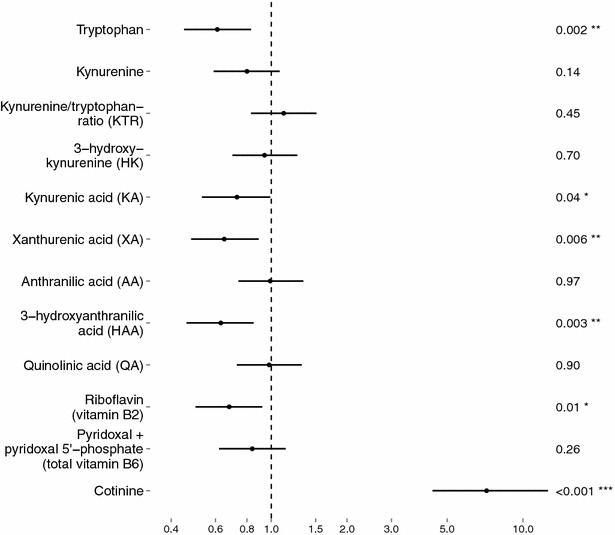
Odds ratio for ADHD with 95 % confidence intervals. Cotinine is divided in three categories corresponding to the serum level in non-smokers, smokers and heavy smokers. High levels of tryptophan, KA, XA, HAA and riboflavin were associated with lower odds ratio for ADHD. High levels of cotinine were associated with higher odds ratio for ADHD. Significance <0.001: ‘***’, <0.01: ‘**’, <0.05: ‘*’

Mann–Whitney U test showed significantly lower serum concentrations in the ADHD group for tryptophan, KA, XA, HAA, vitamin B2 (riboflavin) and total vitamin B6 (PL + PLP), as well as a significantly higher level of cotinine, compared with the control group (Table 1). Distribution of raw biochemical variables in tertiles for patients and controls are shown in Fig. 2. OR with 95 % confidence interval (CI) for ADHD was calculated per tertile/category of each metabolite (Fig. 3; Table 3), using two models: one adjusted for sex, and one adjusted for sex and tryptophan. Adjusting for sex, lower levels of tryptophan, KA, XA, HAA and vitamin B2 were associated with increased risk of having ADHD. In addition, higher levels of cotinine were strongly associated with ADHD. In the second model, adjusting for sex and tryptophan, only HAA, riboflavin and cotinine remained significant predictors of ADHD/control status, while KA and XA were no longer significant.

**Table 3:** Logistic regression

	Adjusted for sex			Adjusted for sex and tryptophan		
	B	p value	Odds ratio (95 % confidence interval)	B	p value	Odds ratio (95 % confidence interval)
Tryptophan	−0.50	0.002**	0.61 (0.45–0.83)			
Kynurenine	−0.23	0.14	0.80 (0.59–1.08)	−0.04	0.80	0.96 (0.69–1.34)
Kyn/Trp-ratio (KTR)	0.12	0.45	1.12 (0.83–1.51)	−0.11	0.53	0.90 (0.64–1.25)
3-hydroxykynurenine (HK)	−0.01	0.70	0.94 (0.70–1.27)	0.01	0.94	1.01 (0.74–1.38)
Kynurenic acid (KA)	−0.32	0.04*	0.73 (0.53–0.99)	−0.21	0.21	0.81 (0.59–1.12)
Xanthurenic acid (XA)	−0.43	0.006**	0.65 (0.48–0.89)	−0.30	0.07	0.74 (0.54–1.03)
Anthranilic acid (AA)	−0.01	0.97	0.99 (0.74–1.34)	0.04	0.79	1.04 (0.77–1.42)
3-hydroxyanthranilic acid (HAA)	−0.47	0.003**	0.63 (0.46–0.85)	−0.38	0.02*	0.69 (0.50–0.94)
Quinolinic acid (QA)	−0.02	0.90	0.98 (0.73–1.32)	0.04	0.82	1.04 (0.76–1.41)
Riboflavin (vitamin B2)	−0.39	0.01*	0.68 (0.50–0.92)	−0.34	0.03*	0.71 (0.52–0.97)
Total vitamin B6	−0.17	0.26	0.84 (0.62–1.14)	−0.09	0.55	0.91 (0.67–1.24)
Cotinine	1.97	<0.001***	7.17 (4.37–12.58)	1.94	<0.001***	6.96 (4.22–12.25)

Odds ratio of ADHD per tertile of variable. Cotinine is divided into three categories corresponding to the serum level in non-smokers, smokers and heavy smokers. All participants included: N = 264. Significance <0.001: ‘***’, <0.01: ‘**’, <0.05: ‘*’

#### Metabolite levels in relation to age, comorbidity and medication

Logistic regression using two age groups of ADHD patients, i.e. 19–33 years and 34–40 years, as outcome were performed to investigate the effect of age. Lower levels of tryptophan [OR: 0.58 (95 % CI 0.35–0.93, p value: 0.03)] and XA [0.56 (0.22–0.91, 0.02)] as well as higher levels of riboflavin [2.13 (1.32–3.54, 0.003)] and higher KTR [1.65 (1.02–2.71, 0.04)] were significantly associated with the age 34–40 group (N = 36). When the age 19–33 ADHD subgroup (N = 97) was compared to the control group (18–33, N = 131), lower levels of tryptophan [0.71 (0.51–0.99, 0.05)], HAA [0.66 (0.47–0.91, 0.01)] and riboflavin [0.53 (0.37–0.75, <0.001)], and higher levels of cotinine [7.84 (4.63–14.22, <0.001)] were significantly associated with increased risk of having ADHD. No significant differences in serum concentrations were found between ADHD patients with or without self-reported anxiety/depression (all p values >0.13) or bipolar disorder (all p values >0.09). Comparisons of levels of tryptophan metabolites between medicated and drug-naïve patients showed a trend towards lower levels of KA and HAA in non-medicated patients in preliminary analyses. However, logistic regression analyses did not yield any significant differences. The number of patients currently using antidepressants was too small to allow for comparisons.

#### Metabolite levels according to symptom scores

Correlation analyses adjusted for cotinine and age showed significant inverse correlations between the serum concentration of tryptophan and kynurenine and the current and past ADHD symptom scores ASRS and WURS, when including all participants (N = 264) (Table 4). Analyses on symptom scores in the ADHD group separately (N = 133) confirmed that lower levels of tryptophan and total vitamin B6 were correlated with higher total ASRS score and higher score on the ASRS inattentive subscale (Table 4). Lower levels of tryptophan were also correlated with higher WURS score. In the control group (N = 131), there were no significant correlations between tryptophan and ADHD symptom scores (Table 4).

**Table 4:** Spearman’s correlations for ADHD symptom scores and serum variables, adjusted for cotinine and age

		Trp	Kyn	KTR	HK	KA	XA	AA	HAA	QA	Vit. B2	Vit. B6
All												
N = 248	ASRS total score	−0.15*	−0.14*	0.04	0.04	−0.08	0.01	0.04	−0.05	−0.04	−0.13*	−0.06
N = 250	ASRS inattentive	−0.15*	−0.13*	0.06	0.02	−0.08	−0.01	0.06	−0.03	−0.01	−0.06	−0.03
N = 251	ASRS hyperactive/impulsive	−0.14*	−0.16*	0.02	0.03	−0.09	0.01	0.02	−0.07	−0.07	−0.17*	−0.05
N = 243	WURS total score	−0.18*	−0.15*	0.03	−0.01	−0.14*	−0.08	0.05	−0.08	−0.05	−0.09	−0.01
ADHD												
N = 121	ASRS total score	−0.20*	−0.16	0.07	0.11	−0.05	0.01	0.05	−0.01	−0.14	−0.17	−0.22*
N = 122	ASRS inattentive	−0.20*	−0.16	0.08	0.11	−0.03	0.06	0.02	0.07	−0.08	−0.09	−0.19*
N = 123	ASRS hyperactive/impulsive	−0.17	−0.14	0.03	0.09	−0.03	−0.02	0.06	−0.05	−0.12	−0.17	−0.14
N = 115	WURS total score	−0.27*	−0.13	0.13	−0.08	−0.18	−0.16	0.15	−0.09	−0.16	0.05	−0.04
Control												
N = 127	ASRS total score	−0.05	−0.11	0.03	−0.11	−0.12	0.01	−0.05	−0.07	−0.10	−0.14	−0.04
N = 128	ASRS inattentive	0.01	−0.02	0.03	−0.13	−0.10	−0.04	0.02	−0.01	−0.01	−0.07	0.03
N = 128	ASRS hyperactive/impulsive	−0.10	−0.19*	−0.01	−0.09	−0.13	0.05	−0.12	−0.10	−0.17	−0.19*	−0.08
N = 128	WURS total score	−0.14	−0.14	0.03	−0.07	−0.11	−0.07	−0.07	−0.06	−0.08	−0.11	−0.01

The 18-item ASRS (Adult ADHD Self-report Scale) is used to assess current ADHD symptom burden, with nine questions specific to hyperactivity/impulsivity and nine questions specific to inattentiveness. WURS (Wender Utah Rating Scale) consists of 25 items for retrospective assessment of childhood ADHD symptom burden. Significance: <0.05 ‘*’

*Trp* tryptophan, *Kyn* kynurenine, *KTR* kynurenine/tryptophan ratio, *HK* 3-hydroxykynurenine, *KA* kynurenic acid, *XA* xanthurenic acid, *AA* anthranilic acid, *HAA* 3-hydroxyanthranilic acid, *QA* quinolinic acid, *Cot* cotinine

## Discussion

In this study of 133 adult patients with ADHD and 131 adult controls, we found significantly lower concentrations of tryptophan, KA, XA and HAA in the patient group (Table 3). These results are strengthened by the observed inverse correlations between levels of tryptophan and kynurenine and total scores on both ASRS and WURS adjusted for smoking and age (Table 4). Together, our findings suggest a connection between severity of ADHD symptoms and serum levels of tryptophan and tryptophan metabolites. Furthermore, significantly lower levels of riboflavin and higher levels of cotinine were found among the ADHD patients compared to the controls (Table 3). The kynurenine/tryptophan ratio (KTR) was not significantly different between the two groups in any of the analyses, and did not show any correlation to ADHD symptom scores (Table 4). Thus, there is no strong indication of chronic immune activation in the ADHD patients.

Our results are different from an earlier, exploratory study on kynurenines in children with ADHD which reported higher serum levels of tryptophan and lower levels of HK in children with ADHD (N = 35) compared to controls (N = 28) [33–35]. We do not have any clear explanation for these different findings for tryptophan levels. If the inverse relation between age and tryptophan levels in the patient versus control groups in our sample is a true finding, we would expect to find different results in tryptophan metabolite studies of children versus adults with ADHD. However, published studies are still too small and few in number to allow for definite conclusions.

High levels of tryptophan have also been shown in urine from ADHD children along with low HAA/HK ratios which made the authors suggest that this was due to a low activity of B6-dependent enzymes [36]. We observed low levels of B6 in our patient group, though not significantly different from controls. Like the low levels of tryptophan observed in our patients, low B6 concentrations could be a sign of poor nutritional status.

### Levels of tryptophan and kynurenines

#### Tryptophan levels, age and IDO/TDO activity

Differences in tryptophan concentrations between ADHD patients and controls could be a result of an abnormal IDO/TDO activity with increased catabolism through the kynurenine pathway. Increased catabolism of tryptophan to kynurenine has been found in patients with depression [16, 37], schizophrenia [38, 39], women with postpartum depression [40] and women with preeclampsia [19]. Tryptophan has also been shown to decrease with age in response to increased immune activity [12]. Increased IDO and TDO activity may increase KTR and the level of kynurenines. However, we found no significant difference in KTR between patients and controls (Table 3). Instead we observed positive correlations between concentration of tryptophan and kynurenine in both the patient and control groups, indicating a normal conversion of tryptophan to kynurenine (Table 2). Furthermore, the levels of kynurenines in the ADHD group were not elevated, but were either similar to those of the control group (Kyn, HK, AA and QA) or lower than in controls (KA, XA and HAA) (Tables 1, 3).

Age and tryptophan were inversely associated in the patient group, but not in the control group, which had a smaller age span (18–33 years). Because of the strong association between age and patient/control-status, we chose to compare two age subgroups of patients, 19–33 years and 34–40 years, in order to control for the effect of age. Analyses showed that lower levels of tryptophan [OR: 0.58 (95 % CI 0.35–0.93, p value 0.03)] and XA [0.56 (0.22–0.91, 0.02)], and higher KTR [1.65 (1.02–2.71, 0.04)], were associated with the age 34–40 subgroup. However, using ADHD-status as outcome when including only controls and patients in the age 19–33 subgroup, there were still significantly lower levels of tryptophan [OR: 0.71 (95 % CI 0.51–0.99, p: 0.05)] and HAA [0.66 (0.47–0.91, 0.01)] in patients. Furthermore, in the partial correlation analysis, tryptophan and kynurenine were both inversely correlated to ADHD symptom scores, even when adjusting for the effect of smoking and age (Table 4). Thus, although there seems to be an association between higher age, lower tryptophan and increased KTR, low level of tryptophan in the ADHD group cannot be fully explained by higher IDO or TDO activity. It seems likely that the difference in tryptophan levels could be partially due to a relative tryptophan deficiency in the ADHD group compared to the control group.

#### Vitamin B2 and B6 levels

We found significantly lower levels of riboflavin (vitamin B2) in the ADHD group, in addition to lower HAA and XA. Our findings confirm a previous study reporting a positive association of HAA and XA levels to concentrations of PLP and riboflavin [10]. Similarly, plasma levels of HK have been observed to be inversely associated with plasma PLP [41]. We found an inverse correlation between ASRS score and total vitamin B6 level in the ADHD group, adjusted for smoking and age (Table 4). It could be that low levels of total vitamin B6 among patients cause accumulation of HK and decreased levels of the other kynurenine metabolites [36, 42, 43]. This is supported by the fact that HAA was still significantly associated with ADHD status when adjusting for tryptophan levels in logistic regression analyses (Table 3). Analyses with KA and XA as predictors also suggest a difference, though not significant (Table 3).

#### Smoking

The high number of smokers, 38.3 % moderate smokers and 27.8 % heavy smokers, is an important and well-known characteristic of the ADHD group. Tryptophan and kynurenines (except HK) have been found to be inversely associated with smoking [12], and associations have been observed between blood levels of the nicotine metabolite cotinine and KTR [44]. Serum levels of riboflavin and PLP are also known to be decreased in smokers [11]. With 66 % smokers, it is likely that the levels of kynurenines in the ADHD group are affected by smoking either directly or via decreased levels of B-vitamins. Still, we found no strong correlation between cotinine and tryptophan, KTR, kynurenines or vitamins in our material when using Spearman’s correlation analyses (Table 2). Furthermore, there were significant inverse correlations between ADHD symptom scores and tryptophan and kynurenine even when adjusting for smoking and age (Table 4).

### Strengths and limitations

Our study included a relatively large number of participants (N = 264) and many measured metabolites, including riboflavin, total vitamin B6 and cotinine, which are important as they influence tryptophan catabolism. There are still no generally accepted objective measures or biomarkers available for diagnosing ADHD. ASRS and WURS are widely used tools for evaluation of present and past ADHD symptoms. The inclusion of ADHD symptom scores, although self-reported, allowed us to analyse the relationship between ADHD and biochemical markers as continuous variables.

The limitations of our study include base-line differences between the patient and the control groups regarding age, comorbid conditions and the proportion of smokers, the possible effect of central stimulants and uncertainty regarding the quality of some blood samples. In our analyses we have explored the possible effect of these factors.

We had no information on dietary habits or the relationship between food intake and time of blood collection. We are therefore unable to conclude regarding the effect of nutrition upon serum levels of vitamin B2, vitamin B6 and tryptophan.

Comorbid conditions are common in ADHD patients, and some of these conditions, notably depression and bipolar disorder, have been linked to alterations in tryptophan catabolism. While regression analysis did not show any differences between ADHD patients with or without anxiety/depression or between patients with or without bipolar disorder, we cannot rule out the possibility that comorbid conditions also could affect tryptophan catabolism.

Little is known about the effect of central stimulants on tryptophan catabolism. The small number of drug-naïve patients in our study did not allow any real comparison between medicated and non-medicated patients. The trend of lower levels of tryptophan metabolites in non-medicated patients in our study is however in line with a previous exploratory study in children with ADHD, showing a potential normalising effect of methylphenidate on tryptophan levels [36]. If this represents a true effect, our results could be an underestimation of the differences in tryptophan levels between ADHD patients and controls.

Some blood samples were shipped by mail, and we do not have access to detailed information about their transit time before they were stored at −80 °C. Storing serum samples in room temperature may increase the levels of AA and reduce HK and HAA within a few days [31]. Thus, we cannot rule out the possibility that the concentrations of these metabolites were also affected by pre-analytic effects. In contrast, the concentration of tryptophan has been shown to be stable [31].

Lastly, the results of statistical analyses of the different compounds were not adjusted for multiple testing. The main reason for this choice was that since the biochemical variables are non-independent (Fig. 1), correction for all regression analyses would be too conservative. Instead, if calculating Bonferroni correction by dividing the critical significance level by the number of group comparisons performed by logistic regression, i.e. 0.05/7, the threshold of significant would be 0.007. In the main logistic regression analysis using patient/control-status as outcome (Table 3) tryptophan (p = 0.002), XA (p = 0.006) and HAA (p = 0.003) would remain significant when applying this correction. Likewise, controlling for the false-discovery rate (FDR-correction) for the 12 analyses in the main logistic regression model (Table 3) would also yield significant differences for tryptophan (p = 0.01), XA (p = 0.02), HAA (p = 0.01) and riboflavin (p = 0.03).

## Summary and conclusion

We found lower levels of tryptophan, KA, XA and HAA in adult patients with ADHD compared to adult controls. The levels of kynurenines are dependent mainly on the concentration of tryptophan, the rate of conversion by the enzymes TDO and IDO, and the level of circulating riboflavin and PLP. High TDO and IDO activity does not seem to explain these results, since we observed a normal kynurenine/tryptophan ratio (KTR) and generally low levels of both tryptophan and kynurenines in the ADHD group. The patient group also had low levels of riboflavin (vitamin B2) and total vitamin B6 (PL + PLP), something that could affect the balance of kynurenines in favour of HK. The oxidative effect of smoking on circulating B vitamins may be the cause of low levels of riboflavin and total vitamin B6, and possibly also the low level of tryptophan. Still, the level of the nicotine metabolite cotinine was only weakly correlated with tryptophan, the kynurenines and the vitamins. Also, low levels of tryptophan were correlated with high ADHD symptom scores, even when adjusting for smoking and age. This was observed not only when all participants were included but also in the patient group alone. Thus, low levels of tryptophan, KA, XA and HAA seem to be best explained by a deficiency in tryptophan and in vitamin B2 and B6. We cannot, however, exclude that differences in age, smoking habits and comorbid disorders could contribute to the observed differences in levels of tryptophan and kynurenines. Further independent and carefully controlled studies are needed to clarify the relationship of tryptophan catabolism and ADHD.

Difference in nutritional status is a possible explanation of both low levels of tryptophan and B vitamins. As has been observed in other studies [2, 45], it may be that patients with high symptom scores have a more disordered lifestyle and possibly also a poorer nutritional status. There is now increasing interest in nutrition and possible effects on ADHD and related symptoms, and it has been shown that dietary interventions are able to reduce symptom burden in children with ADHD [46, 47]. Signs of low activity in PLP dependent enzymes in ADHD patients suggest that pyridoxine treatment may have an effect [36]. It is possible that also tryptophan supplements could be beneficial for some patients with ADHD, but more studies on larger populations are needed to further investigate the relation between ADHD and tryptophan catabolism.

## Abbreviations

AA: anthranilic acid
ADHD: attention-deficit hyperactivity disorder
ASRS: Adult ADHD Self-Report Scale
CI: confidence interval
HAA: 3-hydroxyanthranilic acid
HK: 3-hydroxykynurenine
IDO: indole 2,3-dioxygenase
KAT: kynurenine aminotransferase
KMO: kynurenine 3-monooxygenase
KTR: kynurenine/tryptophan ratio
Kyn: kynurenine
KA: kynurenic acid
NMDAr: *N*-methyl-d-aspartate receptor
OR: odds ratio
QA: quinolinic acid
TDO: tryptophan 2,3-dioxygenase
Trp: tryptophan
WURS: Wender Utah Rating Scale
XA: xanthurenic acid
